# *In vitro* propagation and genome sequencing of three ‘atypical’ *Ehrlichia ruminantium* isolates

**DOI:** 10.4102/ojvr.v87i1.1769

**Published:** 2020-06-24

**Authors:** Junita Liebenberg, Helena C. Steyn, Antoinette I. Josemans, Erika Faber, Erich Zweygarth

**Affiliations:** 1 Vaccines and Diagnostics Development Programme, ARC-Onderstepoort Veterinary Research, Pretoria, South Africa; 2 Epidemiology, Parasites and Vectors Programme, ARC-Onderstepoort Veterinary Research, Pretoria, South Africa; 3 Department of Veterinary Tropical Diseases, Faculty of Veterinary Science, University of Pretoria, Pretoria, South Africa

**Keywords:** *Ehrlichia ruminantium*, heartwater, *in vitro* isolation, tick cell line, comparative genomics

## Abstract

Three isolates of *Ehrlichia ruminantium* (Kümm 2, Omatjenne and Riverside), the causative agent of heartwater in domestic ruminants, were isolated in *Ixodes scapularis* (IDE8) tick cell cultures using the leukocyte fraction of infected sheep blood. All stocks were successfully propagated in IDE8 cells, whereas initiation attempts using endothelial cell cultures were unsuccessful. Therefore, the new technique should be included in any attempt to isolate field strains of *E. ruminantium* to enhance the probability of getting *E. ruminantium* isolates which might not be initiated in endothelial cells. Draft genome sequences of all three isolates were generated and compared with published genomes. The data confirmed previous phylogenetic studies that these three isolates are genetically very close to each other, but distinct from previously characterised *E. ruminantium* isolates. Genome comparisons indicated that the gene content and genomic synteny were highly conserved, with the exception of the membrane protein families. These findings expand our understanding of the genetic diversity of *E. ruminantium* and confirm the distinct phenotypic and genetic characteristics shared by these three isolates.

## Introduction

The intracellular rickettsial agent *Ehrlichia ruminantium* causes a disease commonly known as heartwater or cowdriosis. It is an infectious, non-contagious disease which affects mainly cattle, sheep, goats and some wild ruminants. It is transmitted by ticks of the genus *Amblyomma* and has been reported from almost all African countries south of the Sahara, from the adjacent islands of the Indian Ocean and Atlantic Ocean (Uilenberg 1983) and from some Caribbean islands (Birnie et al. 1984; Perreau et al. 1980).

The method of choice for *in vitro* isolation of *E. ruminantium* is infection of endothelial cells (Bezuidenhout, Paterson & Barnard 1985), the cell type in which organisms occur in infected animals (Cowdry 1926). However, several isolates (Allsopp et al. 2007; Du Plessis & Kümm 1971; Steyn 2009) have failed to establish in endothelial cell cultures (Bezuidenhout & Brett 1992; Bezuidenhout et al. 1988). Besides endothelial cells, tick cell lines have been used to initiate *E. ruminantium* in *in vitro* cultures and have even allowed the establishment of infection directly from the leukocytes of sheep blood (Zweygarth, Josemans & Steyn 2008). Therefore, attempts were made to isolate ‘atypical’ *E. ruminantium* stocks in tick cells, atypical in the sense that they could not be initiated by using the classical ways of infecting endothelial cells (Bezuidenhout et al. 1985; Byrom et al. 1991).

Since the early reports of the *E. ruminantium* Omatjenne and Kümm stocks, it has been clear that these stocks share many phenotypical and genetic characteristics, but they differ from all other isolates (Allsopp et al. 1997, 2001; Du Plessis 1985, 1990). The Kümm stock was prepared from a goat, which was diagnosed with heartwater, from the Northern Province of South Africa, a heartwater endemic area (Du Plessis & Kümm 1971). Sheep injected with a lymph node suspension from goat developed heartwater symptoms. After more than 100 passages in mice it was still found to be pathogenic in mice, sheep and goats, but non-pathogenic to cattle (Du Plessis 1982). Many attempts were made to culture this organism in endothelial cells; however, it was only established in culture in 2002 using different monocyte cell lines (Zweygarth et al. 2002). It was observed that the Kümm stock comprised two 16S rRNA (16S ribosomal ribonucleic acid) genotypes, a 16S genotype typical of West African isolates (Kümm1) isolated in a canine macrophage-monocyte cell line (DH82) and a 16S genotype identical to *E. ruminantium* (Omatjenne) (Kümm2) isolated in a sheep blood mononuclear cell line (E2).

The Omatjenne genotype originated from the farm Omatjenne in the Otjiwarongo district of Namibia, a heartwater- and *Amblyomma*-free area (Du Plessis 1990). Healthy cattle on this farm reacted positively to *E. ruminantium* antigen using an indirect fluorescent antibody (IFA) test. Subsequently, ticks were collected from cattle on the farm and homogenates of individual ticks injected into mice. The serum of a single mouse, inoculated with homogenate prepared from a *Hyalomma truncatum* tick, tested positive in the IFA test. The original infective agent was non-pathogenic to mice, calves and sheep. Only after passaging through three generations of *A. hebraeum*, it became fatal to sheep and mice (Du Plessis 1990). The organisms observed in brain smears of the sheep closely resembled those of the Kümm stock; fewer colonies of small size compared with those typically observed in animals infected with other *E. ruminantium* isolates.

Both stocks were atypical in that they are highly pathogenic to mice, but apparently non-pathogenic to bovine and could not be cultured in endothelial cells. The Kümm stock was described as atypical in that it infected mouse peritoneal macrophages (Du Plessis 1982). Because of the differences in pathogenicity and anomalous behaviour in cell culture, it was questioned whether the Kümm stock belonged to the species *E. ruminantium* (Du Plessis 1982). Likewise Allsopp et al. (1997) suggested that *E. ruminantium* (Omatjenne) (then *Cowdria ruminantium* [Omatjenne]), not to be confused with *Ehrlichia* sp. (Omatjenne) later renamed *Anaplasma* sp. (Omatjenne), may belong to a different species because of its difference in vector specificity and virulence.

Phylogenetic studies revealed that all *E. ruminantium* stocks analysed routinely grouped into one of two major clades, a West African clade and a southern/eastern African clade, except for Kümm2 and/or Omatjenne that clustered either as a unique group or in one of the major clades (Allsopp & Allsopp 2007; Allsopp et al. 1997, 2001; Steyn 2009; Van Heerden et al. 2004). Even isolates from several other geographical areas of Africa, the Indian Ocean islands and the Caribbean cluster with the southern and eastern African isolates can be included in a worldwide clade (Cangi et al. 2016). All these studies, however, were conducted with a limited number of genes, which do not necessarily allow the identification of recombination events. Furthermore, only small numbers of *E. ruminantium* isolates with different genotypes have been isolated in cell culture, which limits studies to link variation in DNA sequence to phenotypic characteristics. Therefore, there is a need to establish more isolates in cell culture that would enable us to conduct experiments to determine virulence, cross-protection between isolates, and to produce whole genome sequences.

This study reports on the isolation of ‘atypical’ *E. ruminantium* in tick cell cultures. In addition, we generated draft genome sequences of all three ‘atypical’ isolates and determined the differences between them and previously characterised *E. ruminantium* genomes.

## Materials and methods

### Infective agents

Three stocks of *E. ruminantium* were used. The Kümm2 genotype (Zweygarth et al. 2002), which was derived from the Kümm stock (Du Plessis 1982), was originally isolated from a naturally infected goat in Rust de Winter (Limpopo Province, South Africa). The *E. ruminantium* Omatjenne isolate was isolated from a single *H. truncatum* tick collected from a heartwater- and *Amblyomma*-free area of Namibia (Du Plessis 1990). Its complete 16S rDNA (16S ribosomal RNA) sequence was determined and submitted to GenBank^TM^ (accession number *C. ruminantium* [Omatjenne] U03776) (Allsopp et al. 1997). The original inoculums from which the Kümm and Omatjenne stocks were isolated are no longer available and the complete history of the blood stabilates used in this study is unknown. The third isolate was derived from the blood obtained from a sick angora goat from the farm Riverside (26.83°E, -33.45°S; Grahamstown, Eastern Cape Province, South Africa) (Steyn 2009).

### Cell cultures

The tick cell line *Ixodes scapularis* (IDE8), derived from *Ixodes scapularis* embryos (Munderloh et al. 1994) was used. It was propagated in L-15B medium (Munderloh & Kurtti 1989), which was supplemented with 5% heat-inactivated foetal bovine serum (FBS), 10% tryptose phosphate broth, 0.1% bovine lipoprotein concentrate (MP Biomedicals, Santa Ana, CA, United States [US]), 100 IU/mL penicillin and 100 *μ*g/mL streptomycin. The pH was adjusted at approximately 7.0. Infected tick cell cultures were maintained in Dulbecco’s modified Eagle’s medium nutrient mixture Ham F-12 (DME/F-12; Sigma, St. Louis, MO, US; D 0547) containing 15 mM HEPES and 1.2 g/L sodium bicarbonate, further supplemented with 10% (volume per volume [v/v]) heat-inactivated FBS, 2 mM l-glutamine, 100 IU/mL penicillin and 100 *μ*g/mL streptomycin, and are referred to as DF-12.

### Infection of *Ixodes scapularis* (IDE8) cell cultures

Each of the *E. ruminantium* stocks was used to infect Merino sheep by intravenous injection of 5 mL of an infectious blood stabilate. The sheep subsequently developed symptoms associated with *E. ruminantium* infection. The body temperature of each sheep was monitored daily and a blood sample was collected when it exceeded 41.0 °C. Blood collected at the peak febrile reaction was used to initiate cell cultures according to the method described by Zweygarth et al. (2008). Thereafter the sheep were treated with tetracycline.

Blood was collected by venipuncture into sterile Vac-u-test^®^ tubes containing heparin (lithium heparin, 14.3 United States Pharmacopoeia (USP) per mL blood) as anticoagulant and stored in ice. The cooled blood was centrifuged (800 × *g*; 10 min; 4 °C) and the buffy coat collected and washed with cold physiological phosphate-buffered saline (PBS). The buffy coat was re-collected, and the red blood cells were lysed for approximately 30 seconds in 18 mL sterile distilled water followed by the addition of 2 mL of 10-fold concentrated Hanks’ balanced salt solution to restore physiological tonicity. The lysate was centrifuged for 5 min at 290 × *g* at 18 °C. The resulting cell pellet was re-suspended in 5 mL DF-12 and distributed into 25 cm² culture flasks containing IDE8 cells. The cultures were incubated at 32 °C.

### DNA preparation and sequencing

The *E. ruminantium* elementary bodies were isolated from the cell culture material on discontinuous Percoll density gradients (Mahan et al. 1995) and the bacterial DNA was extracted with the DNeasy Blood & Tissue kit (Qiagen, GmbH, Hilden, Germany). The genomes were sequenced using Illumina Nextera paired-end libraries on the Illumina MiSeq and/or HiSeq platforms (Illumina, San Diego, CA, US).

### *De novo* assembly

Sequencing reads were processed and assembled using CLC Genomics Workbench version 7.0 (https://www.qiagenbioinformatics.com/). Default parameters were used for quality trimming, and adapter sequences were removed. Trimmed reads < 50 bp were discarded. Several *de novo* assemblies using different combinations of parameters were performed for each data set. The following stringent parameters were used in all assemblies: mismatch cost, insertion cost and deletion cost of three, and length fraction and similarity fraction = 0.9. We varied the minimum contig length, also whether to use global alignment and whether to perform scaffolding or not. Contigs <500 bp and <10-fold coverage were discarded; the remaining contigs were blasted with the National Center for Biotechnology Information’s (NCBI) nucleotide BLAST (blastn, https://blast.ncbi.nlm.nih.gov/Blast.cgi) to identify and remove contaminating data (phiX, tick, and *Mycoplasma* spp.).

The sequences of the contigs from different CLC assemblies for each data set were compiled and joined in GAP4 (Bonfield, Smith & Staden 1995). Discrepancies between different assemblies of the same data sets were checked and incorrect contig sequences were removed. The resulting contigs were ordered to the Welgevonden strain (CR767821) using progressive Mauve (Darling, Mau & Perna 2010) of Mauve version 2.4.0 (Darling et al. 2004).

### *In silico* multi-locus sequence typing

Multi-locus sequence typing (MLST) loci (*gltA, groEL, lepA, lipA, lipB, secY, sodB* and *sucA*), as described by Adakal et al. (2009), were selected for the analysis. The homologous sequences of the eight genes were identified in the complete genome sequences of Welgevonden (NC_005295.2/CR767821.1) and Gardel (NC_006831.1/CR925677.1) or the GAP4 alignments of incomplete genome sequences. These included Ball3, Mara87/7, Blaauwkrans and Kwanyanga from South Africa (unpublished genomes), and Senegal (NZ_MQUJ00000000.1) and Sankat 430 (BDDN01000001 to BDDN01000183) from West Africa. In addition, orthologous genes from other *Ehrlichia* species, as well as the closely related *Anaplasma* species, were included. These were *E. canis* strain Jake (NC_007354), *E. chaffeensis* strain Arkansas (NC_007799), *E. muris* AS145 (NC_023063), *A. marginale* strain Saint Maries (NC_004842), *A. phagocytophilum* strain HZ (NC_007797), *A. centrale* strain Israel (NC_013532) and *A. ovis* strain Haibei (NZ_CP015994).

The aligned concatenated sequences were analysed in CLC Genomics Workbench 7.0. A maximum likelihood phylogenetic tree (Unweighted Pair Group Method using Arithmetic averages [UPGMA], bootstrap analysis with 100 replicates) was created and pairwise comparisons were performed to illustrate relationship between the *E. ruminantium* isolates and the other *Ehrlichia* and *Anaplasma* species.

### Whole genome comparisons

The ordered contig sequences of each genome were concatenated and submitted to NCBI. Protein-coding genes were annotated using NCBI Prokaryotic Genomes Annotation Pipeline (PGAP) (Tatusova et al. 2016) and the resulting GenBank files were used in whole genome comparisons with the Welgevonden and Gardel sequences. Whole-chromosome alignments were performed locally with Blastall (ftp://ftp.ncbi.nlm.nih.gov/blast/executables/blast+/LATEST/); the tabular view option (-m = 8) allowed visualisation of the alignments in the Artemis Comparison Tool (ACT) programme (Carver et al. 2005). In addition, BLAST Ring Image Generator (BRIG) (Alikhan et al. 2011) was used to compare whole genomes and subsets of genes. We used the locus tags for coding sequence (CDS) from the original annotation and publication (Collins et al. 2005) of the Welgevonden strain of *E. ruminantium*. The corresponding Reference Sequence database (RefSeq) locus tags created subsequently by the NCBI Prokaryotic Genome Annotation Pipeline are listed in Table 1-A1.

### Ethical consideration

Experiments were performed in accordance with the stipulations of the animal ethics committee at ARC-Onderstepoort Veterinary Research and approved by the South African Department of Agriculture, Forestry and Fisheries under Section 20 of the *Animal Disease Act* of 1984.

## Results

### Infection of IDE8 cell cultures

Leukocytes isolated from the blood of infected sheep were used as infective inoculum. All three *E. ruminantium* stocks were established successfully in IDE8 cell cultures by this method (Table 1). The Kümm2 stock was detected in Giemsa-stained smears 28 days after initiation. Infection with the Omatjenne stock was first demonstrated on day 47 post-infection. The Riverside isolate was detected in stained smears in IDE8 cell cultures after 25 days. It was not possible to initiate *in vitro* cultures of any of the three isolates using bovine endothelial cells as host cells (data not shown).

**TABLE 1:** Infection of IDE8 cell cultures using leukocytes from ovine blood isolated from sheep infected with three *E. ruminantium* stocks.

*E. ruminantium* stock	First positive day/days to first subculture/days in culture	Passages
Kümm2	28/31/239	9
Omatjenne	47/72/169	5
Riverside	25/32/295	10

### Genome assembly

The average coverage of all assemblies was greater than 100-fold, and the draft genome sequences of Kümm2, Omatjenne and Riverside isolates comprised 6, 7 and 9 contigs, respectively (Table 2). All contigs of these three genomes were successfully mapped to the reference genome (Figure 1-A1). The total length of the joined contig sequences ranged between 1448 megabases (Mb) and 1455 Mb. The remaining gaps were in repeat regions, including both tandem repeats and dispersed repeats with large repeat units.

**TABLE 2:** Assembly information.

Assembly	Kümm2	Omatjenne	Riverside
**CLC assembly†**			
Average read length after trimming (bp)	195	160	221
Average × coverage	109.6	104.6	134.9
Number of contigs	41	63	44
Average contig length (bp) (range of contig lengths)	35 603(1074–208 441)	23 890(1086–107 642)	32 608(1122–240 823)
Total length (bp)	1 459 740	1 434 720	1 434 795
**GAP4 assembly**			
Number of contigs	6	7	9
Average contig length (bp)	242 475	207 235	160 924
Total length (bp)	1 454 859	1 450 642	1 448 329

bp, base pairs.

† , Information in table represents the CLC assembly with the largest average contig size and the smallest number of contigs.

### *In silico* multi-locus sequence typing analysis

Multi-locus sequence typing was used to illustrate the relationship between different *E. ruminantium* isolates. The South African isolates (Welgevonden, Ball3, Mara87/7, Blaauwkrans and Kwanyanga), as well as the isolate from the Caribbean (Gardel), shared more than 99% identity across the eight genes selected for multi-locus sequence typing (MLST), whereas the West African isolates shared 98% identity with the South African isolates (Table 2-A1). The MLST sequences of the three ‘atypical’ isolates were identical, but had only 88% identity compared with other *E. ruminantium* isolates and were separated into a distinct clade in the phylogenetic tree (Figure 1). The other three species of *Ehrlichia* shared 88% – 89% identity (genetic distances 0.11–0.13), whereas different *Anaplasma* species are more diverse with 68.5% – 92.5% identity (distance of 0.08–0.15). In fact, the genetic distance between *A. marginale* and *A. centrale* (0.08, 92.5% identity) is considerably closer than that between the ‘atypical’ and other *E. ruminantium* isolates (0.12, 88% identity).

**FIGURE 1:**
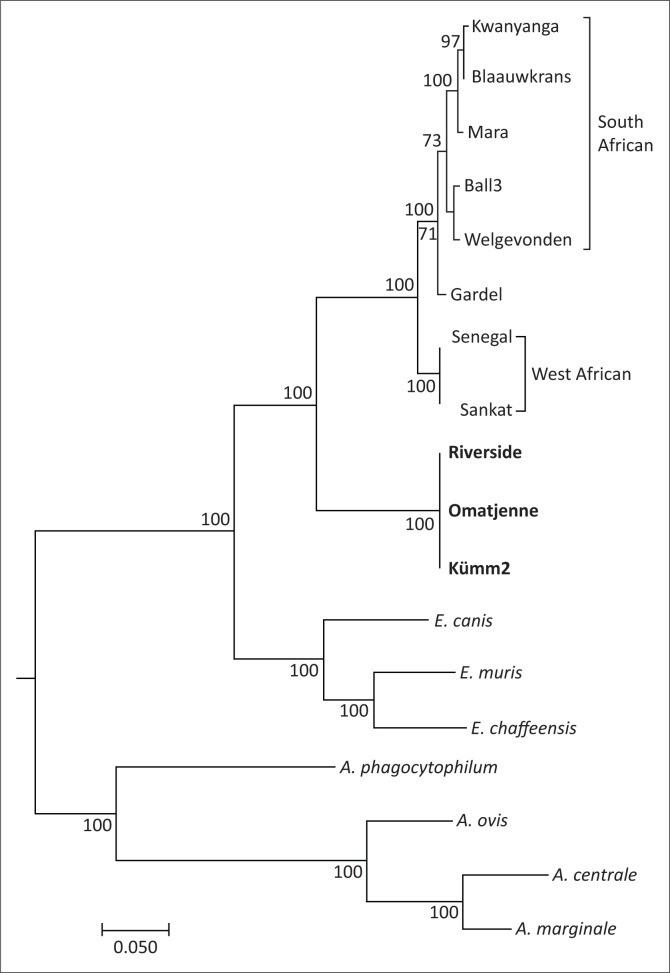
Phylogenetic comparison of the multi-locus sequence typing; Unweighted Pair Group Method using Arithmetic averages tree constructed from the aligned concatenated sequences of the eight selected genes.

### General genome features

All three genomes have a low guanine-cytosine (GC) content (28.94% – 28.95%) and a low protein-coding capacity (65%) (Table 3), which is comparable with the 27.5% GC and 63% – 64% coding capacity of other *E. ruminantium* genomes (Frutos et al. 2006; Nakao, Jongejan & Sugimoto 2016). In each genome, one set of rRNA genes, 36 transfer RNA (tRNA) genes, Transfer-messenger RNA (tmRNA) and two non-coding RNA (ncRNA) genes were identified.

**TABLE 3:** Properties of the annotated genomes.

Isolate	Total length (bp)	GC (%)	No. of CDS	Avg CDS length (bp)	Coding (%)	Pseudo genes	BioSample accession	Genome accession
Kümm2	1455 371	28.95	923	1024	64.9	37	SAMN10340335	CP033456
Omatjenne	1451 340	28.94	925	1018	64.9	32	SAMN10340336	CP033455
Riverside	1449 311	28.94	920	1018	64.6	37	SAMN10340337	CP033454

GC, guanine-cytosine; CDS, coding sequence; bp, base pairs.

The genomes generated in this study still have gaps because of dispersed repeats and large tandem repeats, and certain of the assembled tandem repeats are missing repeat units. These facts could contribute to the smaller genome size (1.45 Mb) of the draft genomes compared with 1.5 Mb reported for the Welgevonden and Gardel sequences (Collins et al. 2005; Frutos et al. 2006). It could also explain the slightly higher protein-coding capacity calculated for the assemblies because the repeat regions are mostly located in non-coding regions.

The gene content and genomic synteny were highly conserved between the three genomes as well as compared with previously sequenced genomes (Figure 2 and Figure 2-A1). Small inversions and rearrangements were found in the area around the shift in GC-skew value between 690 kilobases (kb) and 740 kb as well as other regions where duplications are found (at 630 kb, 1185 kb and 1248 kb) (Figures 1-A1 – 3-A1). A high rate of DNA reorganisation in the terminus region is often observed between closely related bacteria, which may be associated with the mechanism of chromosome separation after replication (Hughes 2000).

**FIGURE 2:**
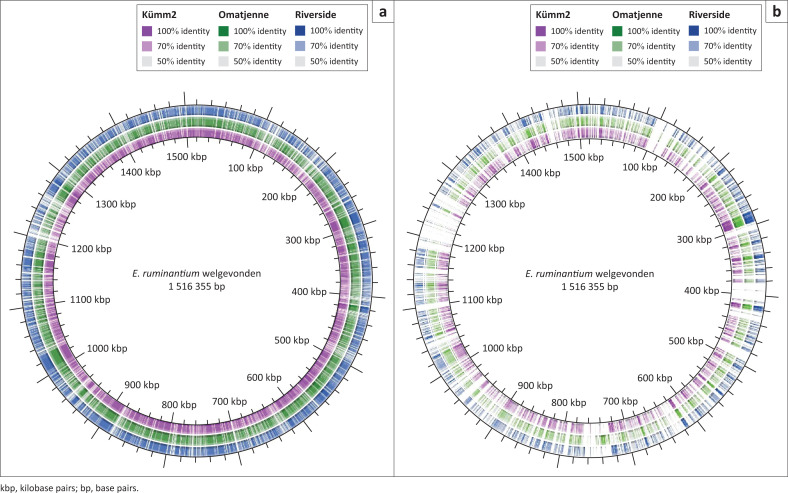
BLAST Ring Image Generator alignments of the three isolates against *E. ruminantium* Welgevonden. (a) Whole genome comparisons of the nucleotide sequence (blastn) and (b) the amino acid sequences comparisons of the coding sequence (blastp).

Single nucleotide polymorphism (SNP) and insert or deletion (INDEL) analysis revealed that when the repeat regions were excluded, only four SNPs and four INDELs distinguished the Kümm2 and Omatjenne sequences. The Riverside sequence differed from either Kümm2 or Omatjenne by 328 SNPs and 16 INDELs.

### Comparison of membrane protein families

Although gene content and genomic synteny are highly conserved, variation was observed between the genomes generated in this study and those reported previously. The variation was mainly limited to genes predicted to encode hypothetical proteins or membrane proteins and specifically the members of the four membrane protein families described previously (Collins et al. 2005). We identified the orthologous families in the Kümm2, Omatjenne and Riverside genomes, and found that their arrangement and number were identical in all the three genomes sequenced in this study. There were, however, variations compared with other *E. ruminantium* isolates. The major antigenic protein 1 (*map1*) family has been described in various isolates. The nucleotide sequences of the members of *map1* family in Kümm2, Omatjenne and Riverside show a high degree of similarity to Welgevonden (Figure 3a), except for *map1-5*. In the syntenic locus of *map1-5*, two smaller open reading frames (ORFs) were identified in the ‘atypical’ genomes (Figure 3b).

**FIGURE 3:**
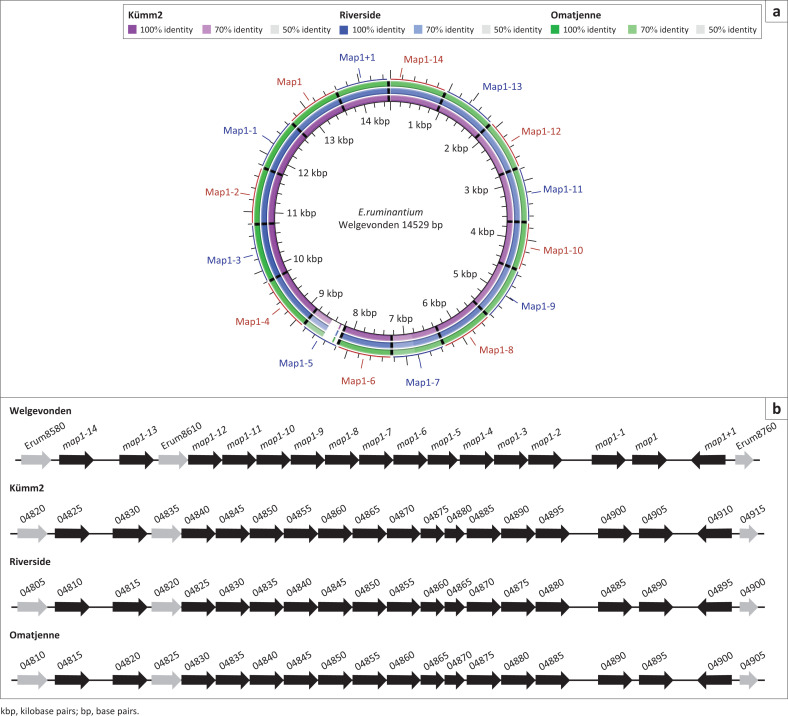
Genomic comparison of the *map1* multigene family. (a) BLAST Ring Image Generator alignments of the three isolates against selected *E. ruminantium* Welgevonden gene sequences. (b) Linear representation of the alignments indicating members of the family in black with adjacent genes in grey. The locus tag prefixes for Kümm2 (EDL79), Riverside (EDL81) and Omatjenne (EDL80) were omitted to simplify the drawing.

Two other families described in Collins et al. (2005), here designated membrane family 1 (Erum2240*–*Erum2340; Erum2400; Erum2410) and membrane family 2 (Erum2750*–*Erum2800; Erum3600*–*Erum3630), were less conserved at nucleotide level (Figure 4a) and both of these families have one member less compared with the Welgevonden annotation (Figure 4b). Three of the genes in membrane family 2 are in opposite orientation in the ‘atypical’ strains as compared with Welgevonden.

**FIGURE 4:**
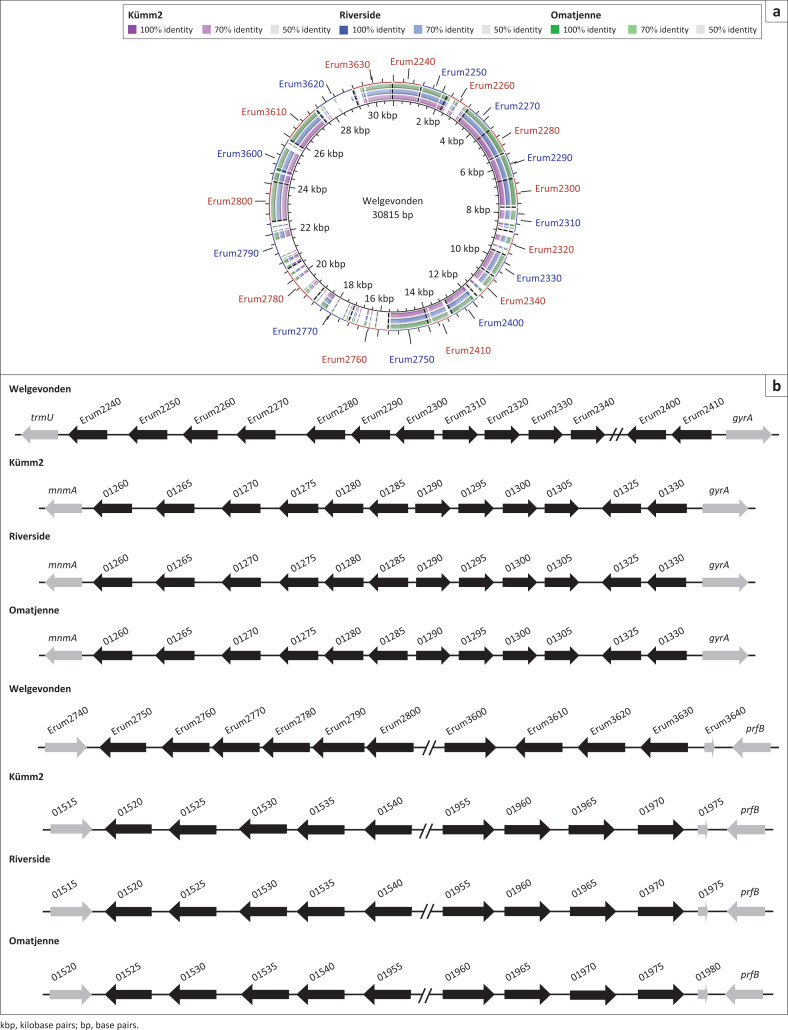
Genomic comparison of membrane families (1) (Erum2240–2410) and (2) (Erum2750–3630). (a) BRIG alignments of the three isolates against selected *E. ruminantium* Welgevonden gene sequences. (b) Linear representation of the alignments indicating members of the families in black with adjacent genes in grey. The locus tag prefixes for Kümm2 (EDL79), Riverside (EDL81) and Omatjenne (EDL80) were omitted to simplify the image.

Orthologs of four predicted membrane proteins (Erum7990, Erum8000, Erum8010 and Erum8020; membrane family 3) were identified in the same relative location in the three newly sequenced genomes (Figure 4-A1). In all three genomes at this location, however, membrane family 3 expanded to seven members and five more members were identified at 82 Mb upstream (Figure 4-A1, Table 3-A1).

## Discussion

*In vitro* isolation of heartwater organisms in cell culture is undoubtedly the most valuable aid for research in heartwater. For years, *E. ruminantium* was isolated from the blood or plasma of reacting animals by cultivation in ruminant endothelial cells, although it was recognised quite early that it was not possible to initiate *in vitro* cultures of some strains using this approach (Bezuidenhout & Brett 1992; Bezuidenhout et al. 1988).

It was shown in previous experiments that leukocytes obtained from four sheep each of which was infected with a different stock of *E. ruminantium* were able to infect IDE8 tick cell cultures and endothelial cell cultures simultaneously (Zweygarth et al. 2008). We applied this technique to the three ‘atypical’ strains that could not be isolated in endothelial cells, the Kümm (Du Plessis 1982), Omatjenne (Du Plessis 1990) and Riverside (Steyn 2009) isolates. Here we show that all three isolates could be established in IDE8 tick cell cultures and continuously propagated for 169 days or more. These cultures provided excess material for additional molecular characterisations of the isolates.

The most distinctive characteristic of the ‘atypical’ isolates is the fact that they cannot be initiated in ruminant endothelial cell cultures. However, some of the reported anomalies may be contributed to other experimental factors. The experiments were conducted using uncharacterised inoculums passaged many times in different animals during the pre-PCR era when it was difficult to detect pathogen-free animals. Hence, the original Kümm stock comprised two organisms or one of them was introduced over the years during passaging. There is also a possibility that the initial Omatjenne agent is not the same as the organism we have cultured in this study. The pathogenicity and vector specificity of the cultured organisms need to be verified experimentally.

The sequences of the concatenated MLST loci of the three ‘atypical’ isolates were identical; however, they formed a distinct clade in tree topology. These results were confirmed by genome sequences. Although these three sequences only differed by a few SNPs and INDELs, as well as variation and small gaps in repeat regions that may be ascribed to assembly errors, they are markedly different from other *E. ruminantium* genomes. The 16S rRNA and *map1* gene sequences identified the Omatjenne agent, and later Kümm2, as *E. ruminantium* in the southern African clade (Allsopp et al. 1997; Van Heerden et al. 2004). In contrast, tree topology and pairwise comparison of eight genes presented in this study may support an argument for the ‘atypical’ isolates to be classified as a separate species. The genetic distances and identity shared between the ‘atypical’ isolates and other *E. ruminantium* isolates are similar to the distances and percentage identity between the different species of *Ehrlichia* and *Anaplasma*. Whether these three isolates indeed represent a unique species needs to be validated.

All the *E. ruminantium* genomes sequenced thus far are syntenic (Frutos et al. 2006; Nakao Jongejan & Sugimoto 2016), and it is known that *Anaplasma* spp. and *Ehrlichia* spp. share conserved gene order (Dunning Hotopp et al. 2006; Pierlé et al. 2012). The synteny is also observed for the three isolates sequenced in this study with a few exceptions in the membrane protein families. Of the four families analysed, the *map1* family was the most conserved one across all *E. ruminantium* genomes. The paralogs are maintained in the same order in all genomes, but in place of *map1-5*, two small ORFs were detected in the genomes presented here. The *map1-5* gene is truncated compared with the other paralogs in all isolates analysed thus far. It was also identified as one of the paralogs that undergoes balancing selection, a type of selection that is reported to maintain genetic variation in genes that are involved in evasion of host immune response (Salim et al. 2019).

Most variation was detected in membrane family 1 and membrane family 2. The nucleotide sequences differed significantly between orthologous genes, and the number, order and, in some cases, orientation of the paralogs were different in new annotations. In membrane family 3, the nucleotide sequences between orthologs were conserved, but this family was expanded from four to 12 members in the ‘atypical’ genomes. At present, the function of these putative proteins is unknown, and it has not been shown that all members of these two protein families are expressed. Therefore, it is not known whether these variations have an effect on the expression or function of the predicted proteins encoded by these ORFs. It is known that *Anaplasma* and *Ehrlichia* spp. present a wide range of paralogous genes encoding various functions that ensure survival in diverse host and vector environments (Dunning Hotopp et al. 2006).

Several studies have reported a high level of genetic diversity among *E. ruminantium* isolates (Allsopp & Allsopp 2007; Cangi et al. 2016; Nakao et al. 2011). In contrast, here we found that the genome sequences of the three ‘atypical’ isolates from distant geographical areas and diverse habitats are almost identical. Excluding repeat regions, Kümm2, from the Limpopo Province of South Africa, and Omatjenne, from the much drier and heartwater-free Otjiwarongo district of Namibia, differ by four SNPs and four INDELs only. In fact, there were more substitutions and small deletions detected between the parental Welgevonden strain and its daughter strain after 11–13 passages in a different cell culture environment (Frutos et al. 2006).

Although the current results have not connected any genetic variation to the phenotypes that distinguish these isolates, variations in the membrane protein families may contribute to the ability of these organisms to infect different cells. A comprehensive SNP analysis, including all genomes sequenced, may elucidate the determinants of diversity. The synteny conservation in *E. ruminantium* genomes suggests that at least some of the phenotypes are associated with small polymorphisms. In view of this, we are in the process of generating whole genome sequences of all the *E. ruminantium* isolates we have in cell culture. In addition, we need to establish more field isolates in culture to conduct phenotypic and genotypic analyses in the future work. To date, no standard cell line has been designated for isolation of *E. ruminantium*, and it is clear that the ‘atypical’ isolates cannot be easily isolated in bovine endothelial cells. We therefore recommend the use of both ruminant endothelial cells and tick cell cultures concurrently. The two methods complement each other and should be used when isolating field strains of *Ehrlichia* spp.

## Figures and Tables

**FIGURE 1-A1:**
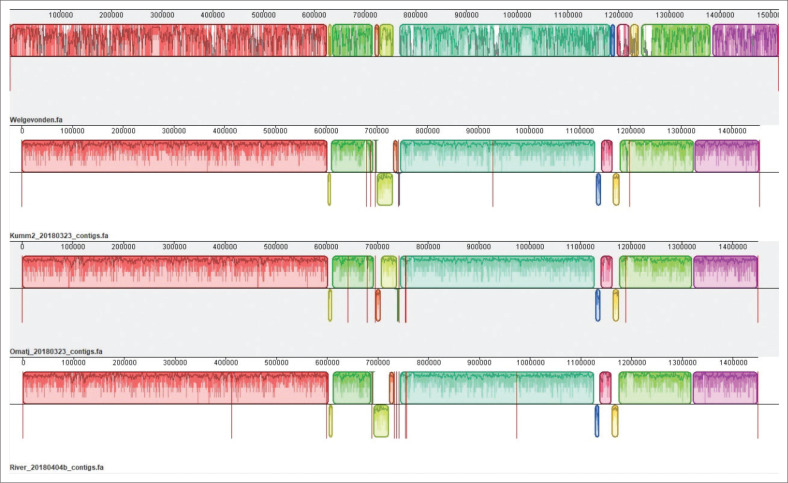
Mauve alignment of the contigs of the three draft genome sequences against the linearised genome sequence of *E. ruminantium* Welgevonden (top panel). The second, third and bottom panels represent the ordered contig sequences, separated with vertical red lines, of Kümm2, Omatjenne and Riverside respectively. Each coloured block represents a segment of the genome sequence that aligned to a homologous part of another genome without any genome rearrangements. Blocks above the centre line of each panel is in the forward orientation relative to the reference genome sequence, while those below the centre line indicate regions that align in the inverse orientation. The height of the similarity profile inside a block corresponds to the average level of conservation in that region.

**FIGURE 2-A1:**
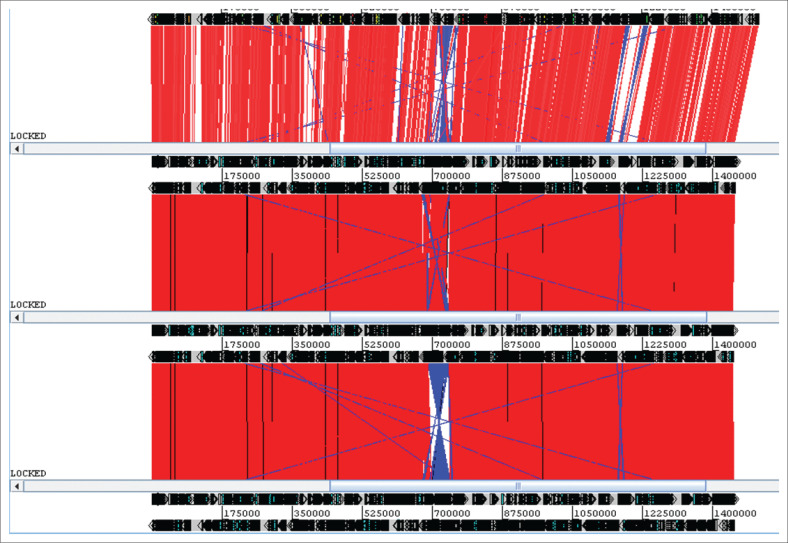
ACT alignment of the three draft genome sequences against the whole genome sequence of *E. ruminantium* Welgevonden. The coloured lines drawn between two adjacent linearised chromosomes show the location of homologous genes and indicate the same (red) or opposite (blue) orientation relative to the chromosome immediately above.

**FIGURE 3-A1:**
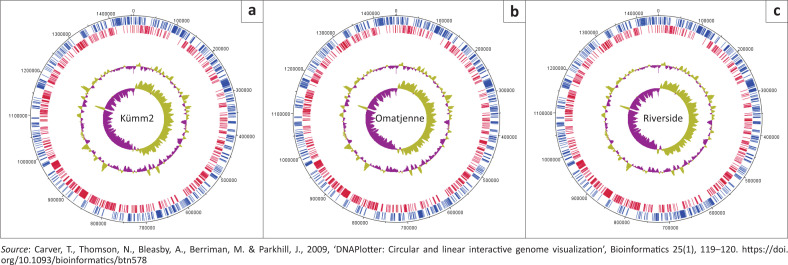
Circular representations of the genomes generated with DNAPlotter. In each circle the tracks from outside to inside represent: (a) coding sequences on the forward (blue) and (b) reverse strands (red), % GC plot, and (c) GC skew [(GC/(G+C)].

**FIGURE 4-A1:**
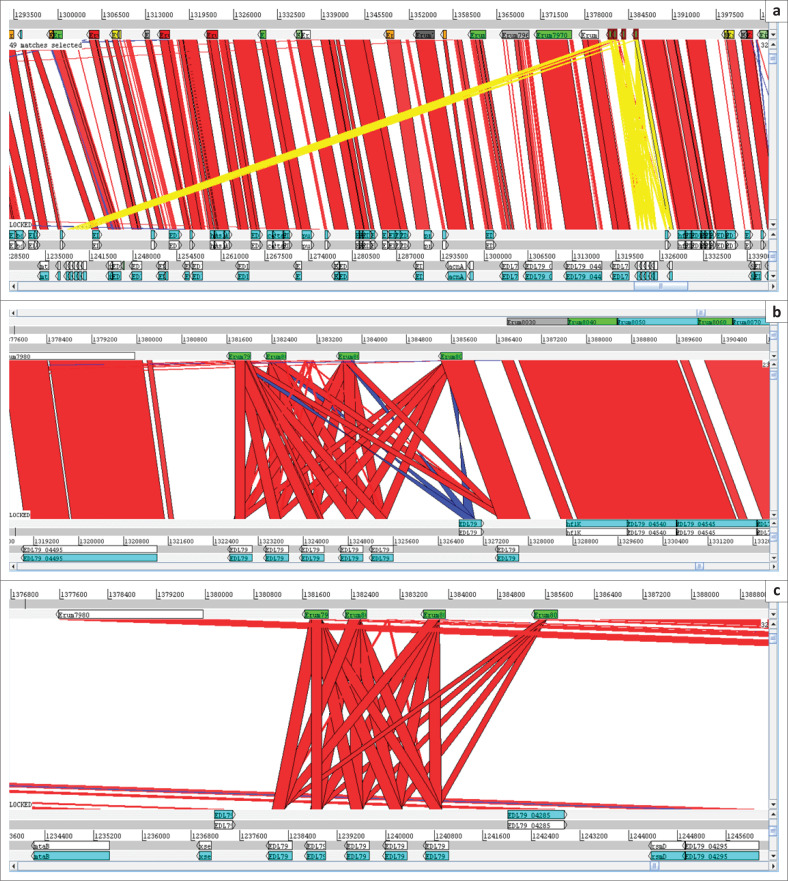
ACT alignment of the *E. ruminantium* Kümm2 sequence (bottom sequence in each panel) against *E. ruminantium* Welgevonden (top sequence in each panel) to depict the expansion of membrane family 3 in the Kümm2 isolate. The coloured lines drawn between the sequences show the location of homologous genes in the same (red) or opposite (blue) orientation. Top (a) Four *E. ruminantium* Welgevonden open reading frames matching open reading frames in two locations (yellow lines) in the *E. ruminantium* Kümm2 annotation. Middle (b) The syntenic matches of the four Welgevonden ORFs to seven Kümm2 open reading frames. Bottom (c) The same four Welgevonden open reading frames matching five Kümm2 open reading frames upstream.

## Appendix 1

**TABLE 1-A1:** The RefSeq locus tags of the members of the membrane protein families.

Membrane protein family	Original annotation			RefSeq locus tags
	ORF ID	Gene name	Functional classification	
**Membrane family 1**	**Erum2230**	***trmU***	**tRNA (5-methylaminomethyl-2-thiouridylate)-methyltransferase**	**ERUM_RS01235**
	Erum2240	-	membrane protein	ERUM_RS01240
	Erum2250	-	membrane protein	ERUM_RS01245
	Erum2260	-	membrane protein	ERUM_RS01250
	Erum2270	-	membrane protein	ERUM_RS01255
	Erum2280	-	membrane protein	ERUM_RS01260
	Erum2290	-	membrane protein	ERUM_RS01265
	Erum2300	-	membrane protein	ERUM_RS01270
	Erum2310	-	membrane protein	ERUM_RS01275
	Erum2320	-	membrane protein	ERUM_RS01280
	Erum2330	-	membrane protein	ERUM_RS01285
	Erum2340	-	membrane protein	ERUM_RS01290
	Erum2400	-	membrane protein	ERUM_RS01320
	Erum2410	-	membrane protein	ERUM_RS01325
	**Erum2420**	***gyrA***	**DNA gyrase subunit A**	**ERUM_RS01330**
**Membrane family 2**	**Erum2740**	**-**	**integral membrane transport protein**	**ERUM_RS01510**
	Erum2750	-	membrane protein	ERUM_RS01515
	Erum2760	-	membrane protein	ERUM_RS01520
	Erum2770	-	membrane protein	ERUM_RS01525
	Erum2780	-	membrane protein	ERUM_RS01530
	Erum2790	-	membrane protein	ERUM_RS01535
	Erum2800	-	membrane protein	ERUM_RS01540
	Erum3600	-	membrane protein	ERUM_RS01960
	Erum3610	-	membrane protein	ERUM_RS01965
	Erum3620	-	membrane protein	ERUM_RS01975
	Erum3630	-	membrane protein	ERUM_RS01980
	**Erum3640**	**-**	**unknown**	**ERUM_RS01985**
**Membrane family 3**	**Erum7570**	**-**	**NADH-ubiquinone oxidoreductase**	**ERUM_RS04090**
	**Erum7580**	**-**	**integral membrane transport protein**	**ERUM_RS04095**
	**Erum7980**	**-**	**type IV secretion system protein**	**ERUM_RS04310**
	Erum7990	-	integral membrane protein	ERUM_RS04315
	Erum8000	-	integral membrane protein	ERUM_RS04320
	Erum8010	-	integral membrane protein	ERUM_RS04325
	Erum8020	-	integral membrane protein	ERUM_RS04330
	**Erum8030**	***hflK***	**FtsH protease activity modulator**	**ERUM_RS04335**
***map1* family**	**Erum8580**	**-**	**transcriptional regulator**	**ERUM_RS04630**
	Erum8590	*map1-14*	outer membrane protein	ERUM_RS04635
	Erum8600	*map1-13*	outer membrane protein	ERUM_RS04640
	**Erum8610**	***-***	**exported protein**	**ERUM_RS04645**
	Erum8620	*map1-12*	outer membrane protein	ERUM_RS04650
	Erum8630	*map1-11*	outer membrane protein	ERUM_RS04655
	Erum8640	*map1-10*	outer membrane protein	ERUM_RS04660
	Erum8650	*map1-9*	outer membrane protein	ERUM_RS04665
	Erum8660	*map1-8*	outer membrane protein	ERUM_RS04670
	Erum8670	*map1-7*	outer membrane protein	ERUM_RS04675
	Erum8680	*map1-6*	outer membrane protein	ERUM_RS04680
	Erum8690	*map1-5*	outer membrane protein	ERUM_RS04685
	Erum8700	*map1-4*	outer membrane protein	ERUM_RS04690
	Erum8710	*map1-3*	outer membrane protein	ERUM_RS04695
	Erum8720	*map1-2*	outer membrane protein	ERUM_RS04700
	Erum8730	*map1-1*	outer membrane protein	ERUM_RS04705
	Erum8740	*map1*	outer membrane protein	ERUM_RS04710
	Erum8750	*map1+1*	outer membrane protein	ERUM_RS04715
	**Erum8760**	**-**	**unknown**	**ERUM_RS04720**

Note: Adjacent genes in bold.

**TABLE 2-A1:** Pairwise comparison table of the multi-locus sequence typing alignment. The upper comparison shows percentage of identical bases in alignment positions between two isolates and the lower comparison depicts the Jukes-Cantor distance between sequences.

*E. ruminantium* isolate	No.	1	2	3	4	5	6	7	8	9	10	11	12	13
Welgevonden	**1**	-	99.56	99.26	99.36	99.35	99.36	97.67	97.70	88.40	88.40	88.40	82.29	81.85
Ball3	**2**	0.00	-	99.28	99.39	99.48	99.36	97.69	97.74	88.41	88.41	88.41	82.25	81.91
Mara	**3**	0.01	0.01	-	99.69	99.60	99.26	97.74	97.77	88.49	88.49	88.49	82.24	81.83
Blaauwkrans	**4**	0.01	0.01	0.00	-	99.90	99.11	97.69	97.74	88.41	88.41	88.41	82.27	81.83
Kwanyanga	**5**	0.01	0.01	0.00	0.00	-	99.12	97.72	97.77	88.44	88.44	88.44	82.28	81.88
Gardel	**6**	0.01	0.01	0.01	0.01	0.01	-	97.87	97.88	88.45	88.45	88.45	82.30	81.89
Senegal	**7**	0.02	0.02	0.02	0.02	0.02	0.02	-	99.94	88.45	88.45	88.45	82.27	81.71
Sankat	**8**	0.02	0.02	0.02	0.02	0.02	0.02	0.00	-	88.46	88.46	88.46	82.27	81.70
Kümm2	**9**	0.12	0.12	0.12	0.12	0.12	0.12	0.12	0.12	-	100	100	82.39	82.11
Omatjenne	**10**	0.12	0.12	0.12	0.12	0.12	0.12	0.12	0.12	0.00	-	100	82.39	82.11
Riverside	**11**	0.12	0.12	0.12	0.12	0.12	0.12	0.12	0.12	0.00	0.00	-	82.39	82.11
*E. canis*	**12**	0.19	0.02	0.02	0.19	0.19	0.19	0.19	0.19	0.19	0.19	0.19	-	87.86
*E. chaffeensis*	**13**	0.02	0.02	0.02	0.02	0.02	0.02	0.02	0.02	0.02	0.02	0.02	0.13	-

**TABLE 3-A1:** Membrane family 3 of the Welgevonden isolate compared to Kümm2, Omatjenne and Riverside.

Welgevonden	Kümm2	Omatjenne	Riverside
1 377 584…1 387 657†	1 319 020…1 329 752	1 315 503…1 326 234	1 312 770…1 323 594
**Erum7980** (**R**‡)	**EDL79_04495 (R)**	**EDL80_04485 (R)**	**EDL81_04475** (R)
	EDL79_04500 (R)	EDL80_04490 (R)	EDL81_04480 (R)
Erum7990 (R)	EDL79_04505 (R)	EDL80_04495 (R)	EDL81_04485 (R)
Erum8000 (R)	EDL79_04510 (R)	EDL80_04500 (R)	EDL81_04490 (R)
Erum8010 (R)	EDL79_04515 (R)	EDL80_04505 (R)	EDL81_04495 (R)
Erum8020 (R)	EDL79_04520 (R)	EDL80_04510 (R)	EDL81_04500 (R)
	EDL79_04525 (F)	EDL80_04515 (F)	EDL81_04505 (F)
	EDL79_04530 (R)	EDL80_04520 (R)	EDL81_04510 (R)
**Erum8030 (*hflK*) (F)**	**EDL79_04535 (*hflK*) (F)**	**EDL80_04525 (*hflK*) (F)**	**EDL81_04515 (*hflK*) (F)**
1 294 686…1 296 536	1 237 185…1 242 924	1 233 416…1 239 155	1 230 696…1 236 436
**Erum7570 (F)**	**EDL79_04255 (F)**	**EDL80_04245 (F)**	**EDL81_04235 (F)**
	EDL79_04260 (R)	EDL80_04250 (R)	EDL81_04240 (R)
	EDL79_04265 (R)	EDL80_04255 (R)	EDL81_04245 (R)
	EDL79_04270 (R)	EDL80_04260 (R)	EDL81_04250 (R)
	EDL79_04275 (R)	EDL80_04265 (R)	EDL81_04255 (R)
	EDL79_04280 (R)	EDL80_04270 (R)	EDL81_04260 (R)
**Erum7580 (F)**	**EDL79_04285 (F)**	**EDL80_04275 (F)**	**EDL81_04265 (F)**

Note: The orthologs flanking the members of the membrane protein family are set in bold.

F, forward strand; R, reverse strand.

† , base location in genome sequence;

‡ , orientation of open reading frame (ORF).

## References

[CIT0001] Adakal H., Meyer D.F., Carasco-Lacombe C., Pinarello V., Allègre F., Huber K. et al., 2009, ‘MLST scheme of *Ehrlichia ruminantium*: Genomic stasis and recombination in strains from Burkina-Faso’, *Infection, Genetics and Evolution* 9(6), 1320–1328. 10.1016/j.meegid.2009.08.003 19712754

[CIT0002] Alikhan N.F., Petty N.K., Ben Zakour N.L. & Beatson S.A., 2011, ‘BLAST Ring Image Generator (BRIG): Simple prokaryote genome comparisons’, *BMC Genomics* 12, 402 10.1186/1471-2164-12-402 21824423 PMC3163573

[CIT0003] Allsopp M.T., Dorfling C.M., Maillard J.C., Bensaid A., Haydon D.T., Van Heerden H. et al., 2001, ‘*Ehrlichia ruminantium* major antigenic protein gene (*map1*) variants are not geographically constrained and show no evidence of having evolved under positive selection pressure’, *Journal of Clinical Microbiology* 39(11), 4200–4203. 10.1128/JCM.39.11.4200-4203.2001 11682561 PMC88518

[CIT0004] Allsopp M.T., Van Strijp M.F., Faber E., Josemans A.I. & Allsopp B.A., 2007, ‘*Ehrlichia ruminantium* variants which do not cause heartwater found in South Africa’, *Veterinary Microbiology* 120(1–2),158–166. 10.1016/j.vetmic.2006.10.026 17123750

[CIT0005] Allsopp M.T.E.P. & Allsopp B.A., 2007, ‘Extensive genetic recombination occurs in the field between different genotypes of *Ehrlichia ruminantium*’, *Veterinary Microbiology* 124(1–2), 58–65. 10.1016/j.vetmic.2007.03.012 17459616

[CIT0006] Allsopp M.T.E.P., Visser E.S., Du Plessis J.L., Vogel S.W. & Allsopp B.A., 1997, ‘Different organisms associated with heartwater as shown by analysis of the 16S ribosomal RNA gene sequences’, *Veterinary Parasitology* 71(4), 283–300. 10.1016/S0304-4017(97)00012-5 9299697

[CIT0007] Bezuidenhout J.D. & Brett S., 1992, ‘Progress with the cultivation of *Cowdria ruminantium* in endothelial cells’, in Dolan T.T. (ed.), *Recent developments in the control of anaplasmosis, babesiosis and cowdriosis*: Proceedings of a Workshop held at ILRAD, Nairobi, Kenya, 13–15 May 1991, pp. 141–147.

[CIT0008] Bezuidenhout J.D., Brett S., Erasmus A. & Rossouw M., 1988, ‘*In vitro* cultivation of *C. ruminantium*’, Biennial Report (1986/87) of the Veterinary Research Institute, Onderstepoort, p. 19.

[CIT0009] Bezuidenhout J.D., Paterson C.L. & Barnard B.J.H., 1985, ‘*In vitro* cultivation of *Cowdria ruminantium*’, *Onderstepoort Journal of Veterinary Research* 52(2), 113–120. 3900854

[CIT0010] Birnie E.F., Burridge M.J., Camus E. & Barré N., 1984, ‘Heartwater in the Caribbean: Isolation of *Cowdria ruminantium* from Antigua’, *The Veterinary Record* 116(5), 121–123. 10.1136/vr.116.5.121 3984174

[CIT0011] Bonfield J.K., Smith K. & Staden R., 1995, ‘A new DNA sequence assembly program’, *Nucleic Acids Research* 23(24), 4992–4999. 10.1093/nar/23.24.4992 8559656 PMC307504

[CIT0012] Byrom B., Yunker C.E., Donovan P.L. & Smith G.E., 1991, ‘*In vitro* isolation of *Cowdria ruminantium* from plasma of infected ruminants’, *Veterinary Microbiology* 26(3), 263–268. 10.1016/0378-1135(91)90019-C 2024445

[CIT0013] Cangi N., Gordon J.L., Bournez L., Pinarello V., Aprelon R., Huber K. et al., 2016, ‘Recombination is a major driving force of genetic diversity in the Anaplasmataceae *Ehrlichia ruminantium*’, *Frontiers in Cellular and Infection Microbiology* 6, 111.eCollection 2016. 10.3389/fcimb.2016.00111 PMC5040723 27747194

[CIT0014] Carver T.J., Rutherford K.M., Berriman M., Rajandream M.A., Barrell B.G. & Parkhill J., 2005, ‘ACT: The Artemis Comparison Tool’, *Bioinformatics* 21(16), 3422–3423. 10.1093/bioinformatics/bti553 15976072

[CIT0015] Carver T., Thomson N., Bleasby A., Berriman M. & Parkhill J., 2009, ‘DNAPlotter: Circular and linear interactive genome visualization’, *Bioinformatics* 25(1), 119–120. 10.1093/bioinformatics/btn578 18990721 PMC2612626

[CIT0016] Collins N.E., Liebenberg J., De Villiers E.P., Brayton K.A., Louw E., Pretorius A. et al., 2005, ‘The genome of the heartwater agent *Ehrlichia ruminantium* contains multiple tandem repeats of actively variable copy number’, *Proceedings of the National Academy of Sciences of the United States of America* 102(3), 838–843. 10.1073/pnas.0406633102 15637156 PMC545511

[CIT0017] Cowdry E.V., 1926, ‘Studies on the aetiology of heartwater: III. The multiplication of *Rickettsia ruminantium* within the endothelial cells of infected animals and their discharge into the circulation’, *Journal of Experimental Medicine* 44(6), 803–814. 10.1084/jem.44.6.803 19869225 PMC2131225

[CIT0018] Darling A.C., Mau B., Blattner F.R. & Perna N.T., 2004, ‘Mauve: Multiple alignment of conserved genomic sequence with rearrangements’, *Genome Research* 14(7), 1394–1403. 10.1101/gr.2289704 15231754 PMC442156

[CIT0019] Darling A.E., Mau B. & Perna N.T., 2010, ‘progressiveMauve: Multiple genome alignment with gene gain, loss and rearrangement’, *PLoS One* 5(6), e11147 10.1371/journal.pone.0011147 20593022 PMC2892488

[CIT0020] Dunning Hotopp J.C., Lin M., Madupu R., Crabtree J., Angiuoli S.V., Eisen J.A. et al., 2006, ‘Comparative genomics of emerging human ehrlichiosis agents’, *PLoS Genetics* 2(2), e21 10.1371/journal.pgen.0020021 16482227 PMC1366493

[CIT0021] Du Plessis J.L., 1982, ‘Mice infected with a *Cowdria ruminantium*-like agent as a model in the study of heartwater’, D.V.Sc thesis, University of Pretoria, South Africa.

[CIT0022] Du Plessis J.L., 1985, ‘A method for determining the *Cowdria ruminantium* infection rate of *Amblyomma hebraeum*: Effects in mice injected with tick homogenates’, *Onderstepoort Journal of Veterinary Research* 52(2), 55–61. 3900855

[CIT0023] Du Plessis J.L., 1990, ‘Increased pathogenicity of an *Ehrlichia*-like organism after passage through *Amblyomma hebraeum*: A preliminary report’, *Onderstepoort Journal of Veterinary Research* 57(4), 233–237. 2293132

[CIT0024] Du Plessis J.L. & Kümm N.A.L., 1971, ‘The passage of *Cowdria ruminantium* in mice’, *Journal of the South African Veterinary Association* 42(3), 217–221. 5161403

[CIT0025] Frutos R., Viari A., Ferraz C., Morgat A., Eychenié S., Kandassamy Y. et al., 2006, ‘Comparative genomic analysis of three strains of *Ehrlichia ruminantium* reveals an active process of genome size plasticity’, *Journal of Bacteriology* 188(7), 2533–2542. 10.1128/JB.188.7.2533-2542.2006 16547041 PMC1428390

[CIT0026] Hughes D., 2000, ‘Evaluating genome dynamics: The constraints on rearrangements within bacterial genomes’, *Genome Biology* 1(6), reviews0006.1–0006.8. 10.1186/gb-2000-1-6-reviews0006 11380986 PMC138892

[CIT0027] Mahan S.M., Andrew H.R., Tebele N., Burridge M.J. & Barbet A.F., 1995, ‘Immunisation of sheep against heartwater with inactivated *Cowdria ruminantium*’, *Research in Veterinary Science* 58(1), 46–49. 10.1016/0034-5288(95)90087-X 7709059

[CIT0028] Munderloh U.G. & Kurtti T.J., 1989, ‘Formulation of medium for tick cell culture’, *Experimental and Applied Acarology* 7(3), 219–229. 10.1007/BF01194061 2766897

[CIT0029] Munderloh U.G., Liu Y., Wang M., Chen C. & Kurtti T.J., 1994, ‘Establishment, maintenance and description of cell lines from the tick *Ixodes scapularis*’, *Journal of Parasitology* 80(4), 533–543. 10.2307/3283188 8064520

[CIT0030] Nakao R., Jongejan F. & Sugimoto C., 2016, ‘Draft genome sequences of three strains of *Ehrlichia ruminantium*, a tick-borne pathogen of ruminants, isolated from Zimbabwe, The Gambia, and Ghana’, *Genome Announcements* 4(3), pii: e00453–16. 10.1128/genomeA.00453-16 27313287 PMC4911466

[CIT0031] Nakao R., Magona J.W., Zhou L., Jongejan F. & Sugimoto C., 2011, ‘Multi-locus sequence typing of *Ehrlichia ruminantium* strains from geographically diverse origins and collected in *Amblyomma variegatum* from Uganda’, *Parasites and Vectors* 4, 137 10.1186/1756-3305-4-137 21762509 PMC3151223

[CIT0032] Perreau P., Morel P.C., Barré N. & Durand P., 1980, ‘Existence de la cowdriose (heartwater) à *Cowdria ruminantium* chez les ruminants des Antilles Françaises (La Guadeloupe) et des Mascareignes (La Reunion et île Maurice)’, *Revue d’Élevage et de Médecine Vétérinaire des Pays Tropicaux* 33(1), 21–22. 7455277

[CIT0033] Pierlé S.A., Dark M.J., Dahmen D., Palmer G.H.& Brayton K.A., 2012, ‘Comparative genomics and transcriptomics of trait-gene association’, *BMC Genomics* 13, 669 10.1186/1471-2164-13-669 23181781 PMC3542260

[CIT0034] Salim B., Amin M., Igarashi M., Ito K., Jongejan F., Katakura K. et al., 2019, ‘Recombination and purifying and balancing selection determine the evolution of major antigenic protein 1 (*map 1*) family genes in *Ehrlichia ruminantium*’, *Gene* 683, 216–224. 10.1016/j.gene.2018.10.028 30316923

[CIT0035] Steyn H.C., 2009, ‘Molecular epidemiology of heartwater (*Ehrlichia ruminantium*) in livestock of rural communities in South Africa’, D.Tech thesis, Tshwane University of Technology, South Africa.

[CIT0036] Tatusova T., DiCuccio M., Badretdin A., Chetvernin V., Nawrocki E.P., Zaslavsky L. et al., 2016, ‘NCBI prokaryotic genome annotation pipeline’, *Nucleic Acids Research* 44(14), 6614–6624. 10.1093/nar/gkw569 27342282 PMC5001611

[CIT0037] Uilenberg G., 1983, ‘Heartwater (*Cowdria ruminantium* infection): Current status’, *Advances in Veterinary Science and Comparative Medicine* 27, 427–480. 6359836

[CIT0038] Van Heerden H., Steyn H.C., Allsopp M.T., Zweygarth E., Josemans A.I. & Allsopp B.A., 2004, ‘Characterization of the pCS20 region of different *Ehrlichia ruminantium* isolates’, *Veterinary Microbiology* 101(4), 279–291. 10.1016/j.vetmic.2004.02.015 15262001

[CIT0039] Zweygarth E., Josemans A.I. & Steyn H.C., 2008, ‘*In vitro* isolation of *Ehrlichia ruminantium* from ovine blood into *Ixodes scapularis* (IDE8) cell cultures’, *Onderstepoort Journal of Veterinary Research* 75(2), 121–126. 10.4102/ojvr.v75i2.10 18788205

[CIT0040] Zweygarth E., Josemans A.I., Van Strijp M.F., Van Heerden H., Allsopp M.T. & Allsopp B.A., 2002, ‘The Kümm isolate of *Ehrlichia ruminantium: In vitro* isolation, propagation and characterization’, *Onderstepoort Journal of Veterinary Research* 69(2), 147–153. 12234001

